# Effect of Long-Term Frozen Storage on Health-Promoting Compounds and Antioxidant Capacity in Baby Mustard

**DOI:** 10.3389/fnut.2021.665482

**Published:** 2021-04-06

**Authors:** Fen Zhang, Jiaqi Zhang, Hongmei Di, Pingxin Xia, Chenlu Zhang, Zihan Wang, Zhiqing Li, Shuya Huang, Mengyao Li, Yi Tang, Ya Luo, Huanxiu Li, Bo Sun

**Affiliations:** ^1^College of Horticulture, Sichuan Agricultural University, Chengdu, China; ^2^Institute of Pomology and Olericulture, Sichuan Agricultural University, Chengdu, China

**Keywords:** baby mustard, long-term frozen storage, blanching, glucosinolates, antioxidants

## Abstract

This study investigated the effects of blanching and subsequent long-term frozen storage on the retention of health-promoting compounds and antioxidant capacity in frozen lateral buds of baby mustard. Results showed that all glucosinolates were well preserved during frozen storage, and 72.48% of total glucosinolate content was retained in the unblanched treatment group after 8 months, as were chlorophylls, carotenoids, ascorbic acid, total phenolics, soluble sugars, soluble proteins, and antioxidant capacity. The loss of nutritional qualities mainly occurred in the 1st month of frozen storage, and nutritional qualities in the unblanched treatment group were significantly better than those in the blanched treatment group during frozen storage. Blanching before freezing reduced contents of high-content glucosinolates and ascorbic acid, as well as antioxidant capacity levels. Therefore, we recommend using long-term frozen storage to preserve the quality of baby mustard to achieve annual supply, and freezing without blanching.

## Introduction

Vegetables are good sources of biologically active components and play an important role in reducing obesity and various diseases (1, 2). Epidemiological studies have provided convincing evidence that the consumption of *Brassica* vegetables is associated with a reduced risk of various cancers and cardiovascular diseases. These health-promoting and anti-carcinogenic properties have been primarily attributed to the high contents of glucosinolates present in *Brassica* vegetables (3–5). Baby mustard (*Brassica juncea* var. *gemmifera*) is a variant of stem mustard and belongs to the *Brassica* genus. Aside from glucosinolates, baby mustard is also a rich source of carotenoids, ascorbic acid, and various phenolics (5, 6), which are also considered important antioxidants because of their activity against free radicals, carcinogenesis, and cardiovascular diseases, as well as in stimulating the human immune system (1, 7, 8). However, the nutritional quality of baby mustard depends on not only the bioactive molecule content when harvested, but also the changes that occur during postharvest handling and storage conditions (1).

Baby mustard is mainly produced in Southwest China and is a seasonal vegetable with a supply period of ~4 months, usually lasting from November to February. It is not available during the other 8 months and is a highly perishable vegetable with a shelf life of on days before it is unsafe or undesirable for consumption (5, 6). Once it is harvested, baby mustard begins to undergo higher rates of respiration, resulting in moisture loss, browning of the epidermis, and quality deterioration (6). These characteristics of baby mustard cause difficulties in its distribution and marketing as fresh produce. Therefore, the production and supply chain of baby mustard needs suitable post-harvest techniques to extend its shelf life and preserve health-promoting compounds.

Many ways of handling vegetables post-harvest have been investigated to extend shelf life and preserve health-promoting compounds, among which frozen storage is one of the most popular and effective processing approaches to maintain post-harvest quality and nutritional properties for extended periods, because at low temperature, deteriorative reactions are reduced to minimal rates (9, 10). According to previous reports, long-term frozen storage is an excellent technology to preserve the post-harvest quality of broccoli florets (11, 12). Likewise, Volden et al. (13) found that frozen cauliflower can serve as an excellent supply of important health-related compounds, even after storage for 1 year. However, in some instances the contents of health-promoting phytochemicals and antioxidant capacity in vegetables are altered during frozen storage (11). In addition, vegetables often require blanching prior to frozen storage in order to inactivate enzymes (14), although some vegetables are frozen without blanching because blanching significantly reduces the nutritional qualities of frozen products (1, 15, 16). Until now, limited information has been available about the retention of nutritional qualities in baby mustard during long-term frozen storage and the blanching process. The current study was, therefore, conducted to investigate the effects of long-term frozen storage and blanching before freezing on contents of health-promoting compounds and the antioxidant capacity of baby mustard lateral buds, and in order to determine whether these methods are a satisfactory way to achieve annual supply of baby mustard.

## Materials and Methods

### Plant Materials

Baby mustard (*Brassica juncea* var. *gemmifera* cv. Linjiang-Ercai) for this study was provided by a local farm in Chengdu City, China. Heads with uniform size and absence of external damage were selected and harvested in the morning and transported to the laboratory immediately. Healthy lateral buds, the main edible parts of baby mustard, were cut off, washed in tap water, dried on blotting paper, and then cut into thin slices of ~3 mm. The lateral buds were assigned randomly to “unblanched” and “blanched” treatment groups.

### Blanching and Storage Treatments

The blanched treatment group of bud slices was randomly divided into four samples that were separately blanched at 96°C for 30 s and then immediately cooled in cold water (3°C for 90 s), drained, and blotted dry using paper towels. Similarly, the unblanched treatment group of bud slices were also randomly divided into four samples and each sample was divided into six portions (300–350 g each) that were packed into transparent polypropylene containers with lids, and then stored in a domestic deep chest freezer at −20°C. The test lasted 8 months and the baby mustard was sampled after 0, 1, 2, 4, 6, and 8 months of storage. The samples were then lyophilized in a freeze-dryer and stored at −20°C for further analysis.

### Quality Assessment

#### Glucosinolate Composition and Contents

Glucosinolates were extracted and analyzed as previously described (17). Freeze-dried samples (100 mg) were boiled in 5 mL water for 10 min. The supernatant was collected after centrifugation, and the residues were washed once with water, centrifuged and then combined with the previous extract. The aqueous extract was applied to a DEAE-Sephadex A-25 column (Sigma Chemical Co., Saint Louis, USA). The glucosinolates were converted into their desulpho analogs by overnight treatment with 100 μL of 0.1% aryl sulphatase (Sigma Chemical Co., Saint Louis, USA), and the desulphoglucosinolates were eluted with 1 mL water. High performance liquid chromatography (HPLC) analysis of desulphoglucosinolates was carried out using an Agilent 1260 HPLC instrument equipped with a variable wavelength detector (VWD) detector (Agilent Technologies, Inc., Palo Alto, USA). Samples were separated at 30°C on a Waters Spherisorb C18 column (250 mm × 4.6 mm i.d.; 5 μm particle size) using acetonitrile and water at a flow rate of 1.0 mL min^−1^. Absorbance was detected at 226 nm. Glucosinolates were quantified by using *ortho*-Nitrophenyl β-D-galactopyranoside (Sigma Chemical Co., Saint Louis, USA) as the internal standard and considering the response factor of each glucosinolate.

#### Chlorophyll and Carotenoid Contents

The contents of chlorophyll and carotenoid were determined using the method of Sun et al. (6). Two hundred mg powder of lateral bud were ground and extracted with 25 mL acetone. The samples were sonicated for 20 min, and centrifuged at 4,000 g at room temperature (20 ± 2°C) for 5 min. The supernatant was filtered through 0.22 μm nylon syringe filters and analyzed by HPLC. HPLC analysis of chlorophylls and carotenoids were carried out using an Agilent 1260 instrument with a VWD detector (Agilent Technologies, Inc., Palo Alto, USA). Samples (10 μL) were separated at 30°C on a Waters C18 column (150 mm × 3.9 mm i.d.; 4 μm particle size) using isopropanol and 80% acetonitrile-water at a flow rate of 0.5 mL min^−1^. Absorbances were detected at 448 and 428 nm. Chlorophylls (a and b) and carotenoids (neoxanthin, violaxanthin, lutein, and β-carotene) were quantified according to the respective standard calibration curves, and their standards were obtained from Solarbio Science and Technology Co., Ltd. (Beijing, China).

#### Ascorbic Acid Content

Ascorbic acid content was determined according to the previous report (17). Fifty mg of sample powder was extracted with 5 mL 1.0% oxalic acid, subsequently centrifuged 5 min at 4,000 g. Each sample was filtered through a 0.45 μm cellulose acetate filter. HPLC analysis of ascorbic acid was carried out using an Agilent 1260 instrument with a VWD detector (Agilent Technologies, Inc., Palo Alto, USA). Samples were separated on a Waters Spherisorb C18 column (150 mm × 4.6 mm i.d.; 5 μm particle size), using a solvent of 0.1% oxalic acid at a flow rate of 1.0 mL min^−1^. The amount of ascorbic acid was calculated from absorbance values at 243 nm, using authentic ascorbic acid (Sangon Biotech Co., Ltd., shanghai, China) as a standard.

#### Total Phenolics Content

Total phenolics were homogenized for 1 min and extracted with 10 mL of 50% ethanol, and then incubated at room temperature (20 ± 2°C) for 24 h in the dark. The suspension was centrifuged at 4,000 g for 5 min at room temperature. The supernatant was used for the measurements of total phenolics content and antioxidant activity. The supernatant was mixed with Folin-Ciocalteu reagent, after 3 min, saturated sodium carbonate was added. The absorbance was measured at 760 nm with a UV-1800 spectrophotometer (Mapada Instruments Co., Ltd., Shanghai, China) as previously described (17). Gallic acid (Sangon Biotech Co., Ltd., shanghai, China) was used as a standard and the results were expressed as mg gallic acid equivalent g^−1^ dry weight.

#### Ferric Reducing Antioxidant Power (FRAP)

FRAP assay was performed according to the previous report (17). The extracted samples were added to the FRAP working solution incubated at 37°C and vortexed. The absorbance was then recorded at 593 nm using a UV-1800 spectrophotometer (Mapada Instruments Co., Ltd., Shanghai, China) after the mixture had been incubated in at 37°C for 10 min. FRAP values were calculated based on FeSO_4_·7H_2_O standard curves and expressed as μmol g^−1^ dry weight.

#### 2,2-Azinobis (3-Ethyl-Benzothiazoline-6-Sulfonic Acid) (ABTS) Assay

ABTS antioxidant activity was performed according to the previous report (17). An aliquot of 300 μL of each extracted sample was added to 3 mL of ABTS^+^ solution. The absorbance was measured spectrophotometrically at 734 nm after exactly 2 h. The percentage inhibition was calculated according to the formula: % inhibition = [(A_control_-A_sample_)/A_control_] × 100%.

#### Soluble Sugar Composition and Contents

Soluble sugars, including fructose, glucose, and sucrose, were extracted and analyzed as previously described with some modification (6). Freeze-dried samples (100 mg) were added to 5 mL of distilled water and homogenized for 1 min. The mixture was then extracted in a water bath at 80°C for 30 min. The supernatant was collected after centrifugation at 8,000 g at room temperature (20 ± 2°C) for 5 min, and filtered through 0.45 μm cellulose acetate filter, and then analyzed by HPLC using an Agilent 1260 instrument equipped with a refractive index detector (Agilent Technologies, Inc., Palo Alto, USA). Samples were separated at 35°C on an Agilent ZORBAX carbohydrate column (250 mm × 4.6 mm i.d.; 5 μm particle size) using 80% acetonitrile at a flow rate of 1.0 mL min^−1^.

#### Soluble Proteins Content

The soluble proteins content was determined using the method of Bradford (18). Fifty milligrams of freeze-dried powdered material was soaked in 10 mL of distilled water. The solution was stirred for 30 s using a vortex mixer, after which it was allowed to settle for 30 min. The solution was then centrifuged for 5 min at 4,000 g and 1 mL transferred to a polypropylene tube. Subsequently, Coomassie brilliant blue G-250 was combined with 1 mL of supernatant. The absorbance was measured at 595 nm within 20 min after the reaction.

### Statistical Analysis

All assays were performed in quadruplicate. Statistical analysis was performed using the SPSS package program version 18 (SPSS Inc., Chicago, IL, USA). Data were analyzed using two-way analysis of variance. Principal component analysis (PCA) was performed in SIMCA-P 11.5 Demo software (Umetrics, Sweden) with unit variance (UV)-scaling to decipher the relationships among samples (17). A time-related trajectory analysis based on two-dimensional PCA map was applied to visualize the temporal alterations of postharvest quality changes under different photoperiod treatments (6).

## Results

### Glucosinolates

**Figure 1:**
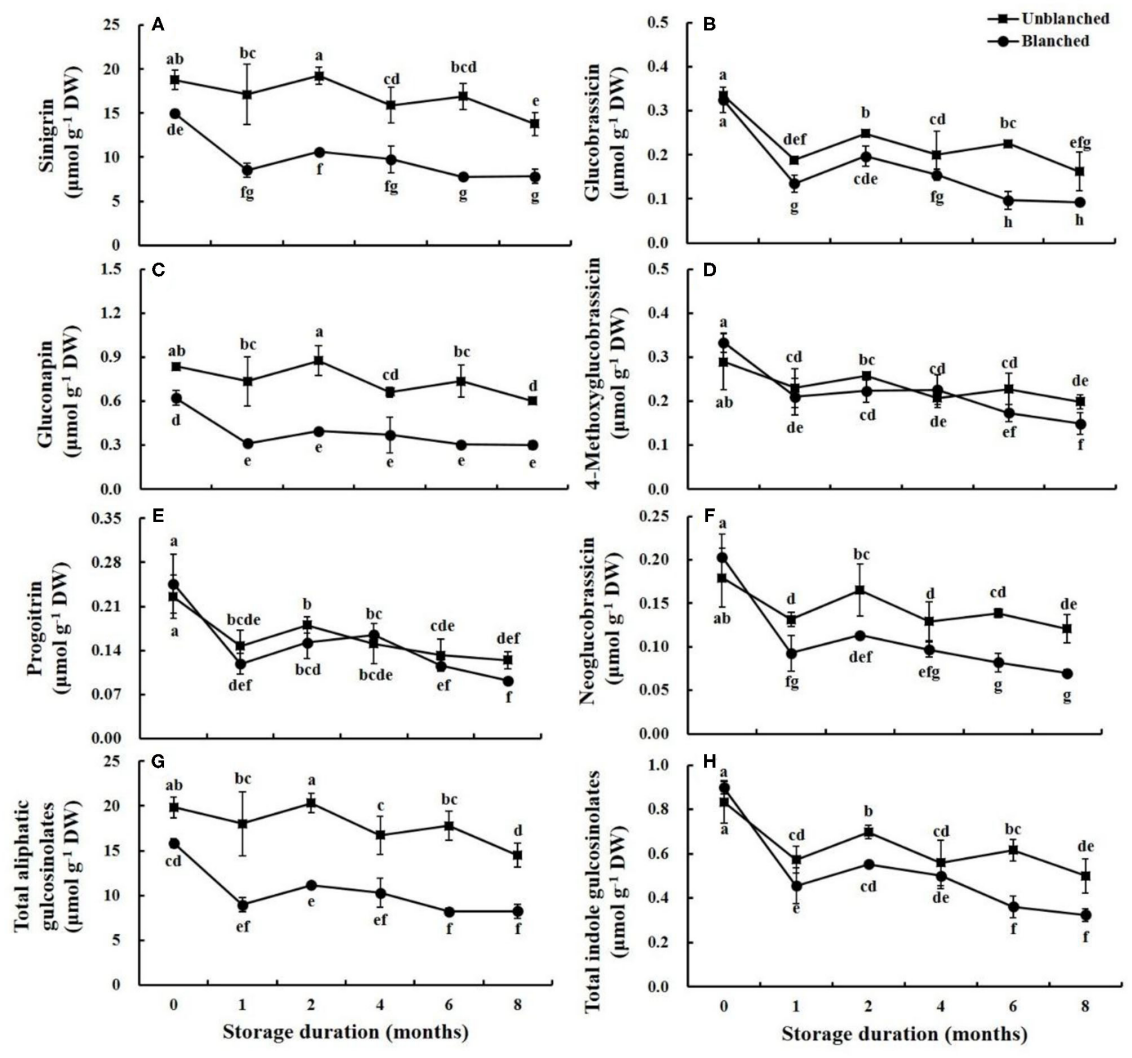
Evolution trend of predominant glucosinolate content in baby mustard lateral buds during frozen storage at −20°C. **(A)** sinigrin; **(B)** glucobrassicin; **(C)** gluconapin; **(D)** 4-methoxyglucobrassicin; **(E)** progoitrin; **(F)** neoglucobrassicin; **(G)** total aliphatic gulcosinolates; **(H)** total indole gulcosinolates. Unblanched represents no blanching before freezing; Blanched represents blanching before freezing. Values not sharing the same letter are significantly different at *p* < 0.05.

The composition and contents of glucosinolate in the lateral buds of baby mustard were measured, including three aliphatic glucosinolates (sinigrin, gluconapin, and progoitrin) and four indole glucosinolates (glucobrassicin, 4-methoxyglucobrassicin, neoglucobrassicin, and 4-hydroxy glucobrassicin) (Figure 1 and Supplementary Figure 1). The most abundant glucosinolate was sinigrin, accounting for 94.65 and 90.84% of total aliphatic and total glucosinolate contents, respectively, followed by gluconapin. The predominant indole glucosinolates were glucobrassicin and 4-methoxyglucobrassicin.

After blanching, the contents of sinigrin, gluconapin, total aliphatic glucosinolate, and total glucosinolate decreased by 20.34, 25.59, 20.22, and 19.09%, respectively compared with unblanched buds. On the other hand, the contents of progoitrin and indole glucosinolates (except for glucobrassicin) slightly increased, while glucobrassicin content remained stable after blanching (Figure 1 and Supplementary Figure 1).

During long-term frozen storage, the contents of individual and total aliphatic glucosinolates (except for progoitrin) in the unblanched treatment group showed a slow, wavelike decline, while the contents in the blanched treatment group decreased rapidly in the 1st month and then remained stable (Figure 1). Due to the large proportion of sinigrin, the trend of total glucosinolate content was similar to that of sinigrin (Supplementary Figure 1). The contents of individual and total indole glucosinolates, as well as progoitrin, decreased slightly after a sharp decline in the 1st month of frozen storage regardless of blanching status (Figure 1 and Supplementary Figure 1). Moreover, the contents of most glucosinolates in the unblanched treatment group were significantly higher than those in the blanched treatment group during frozen storage. For example, contents of sinigrin and total glucosinolate in the unblanched treatment group were 1.76- and 1.75-fold higher than those in the blanched treatment group after 8 months, respectively. Overall, however, at the end of storage, the total glucosinolate contents in unblanched and blanched treatment groups had decreased by 27.52 and 58.70% compared with respective initial levels, suggesting that long-term frozen storage could be a good way to preserve the glucosinolates in frozen baby mustard.

### Chlorophylls and Carotenoids

**Figure 2:**
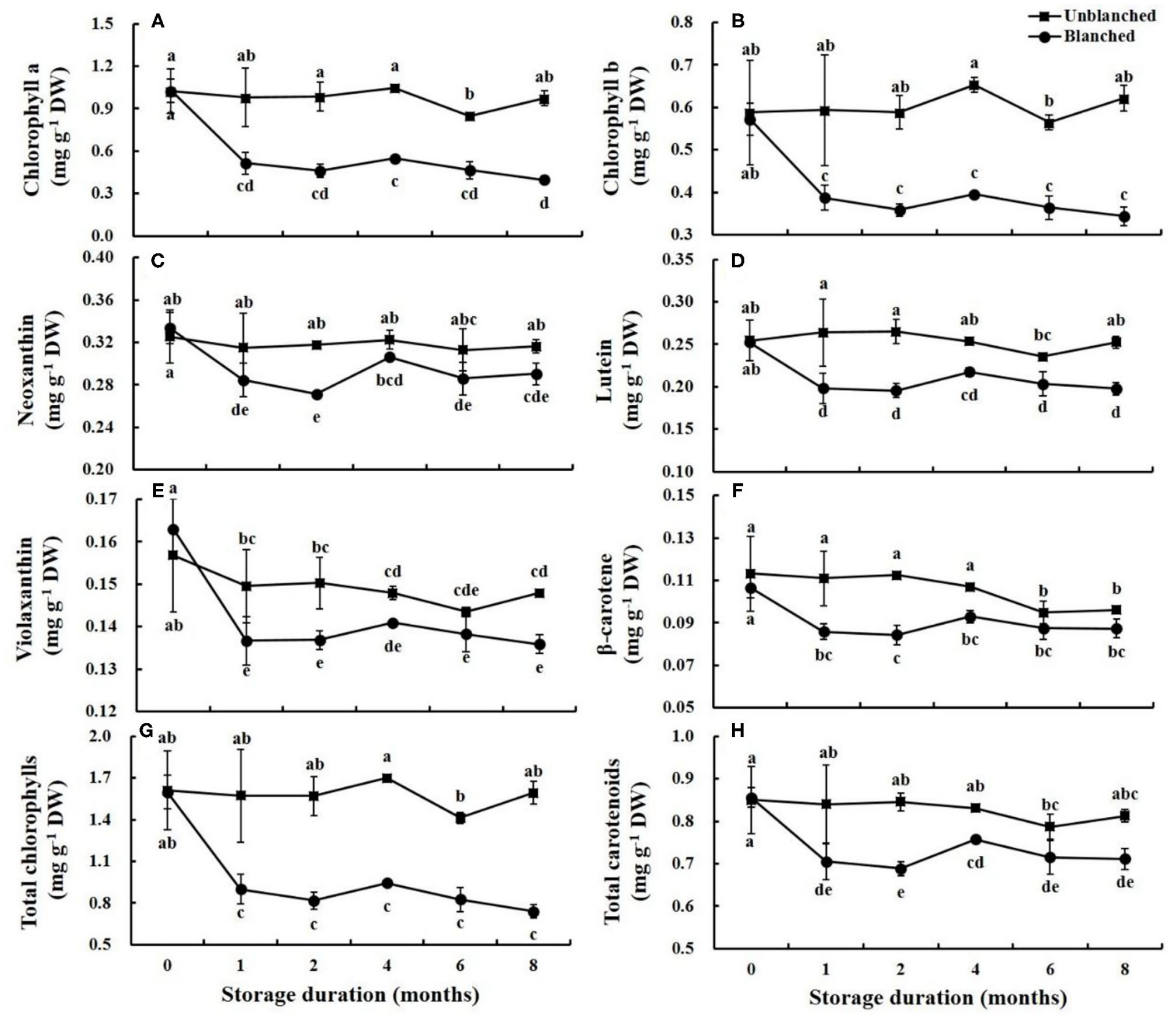
Evolution trend of chlorophylls and carotenoids contents in baby mustard lateral buds during frozen storage at −20°C. **(A)** chlorophyll a; **(B)** chlorophyll b; **(C)** neoxanthin; **(D)** lutein; **(E)** violaxanthin; **(F)** β-carotene; **(G)** total chlorophylls; H: total carotenoids. Unblanched represents no blanching before freezing; Blanched represents blanching before freezing. Values not sharing the same letter are significantly different at *p* < 0.05.

There were two chlorophylls (chlorophyll a and chlorophyll b) and four carotenoids (neoxanthin, violaxanthin, lutein, and β-carotene) detected in the baby mustard buds (Figure 2). Blanching before freezing did not have a significant effect on the chlorophyll and carotenoid contents in the buds. During long-term frozen storage, contents of chlorophyll and carotenoid in lateral buds in the unblanched treatment group remained stable, whereas contents in the blanched treatment group decreased rapidly in the 1st month and then remained stable, and the total chlorophyll and carotenoid contents in the blanched treatment group decreased by 54.03 and 16.32% at 8 months, respectively. Most of the individual and total chlorophyll and carotenoid contents in the unblanched treatment group were significantly higher than those in the blanched treatment group during frozen storage, and contents of total chlorophylls and carotenoids in unblanched buds were 2.15- and 1.14-fold those in blanched buds at the end of storage, respectively. In general, the chlorophyll and carotenoid contents of lateral buds were effectively preserved by long-term frozen storage, especially in the unblanched treatment group.

### Ascorbic Acid and Total Phenolics

**Figure 3:**
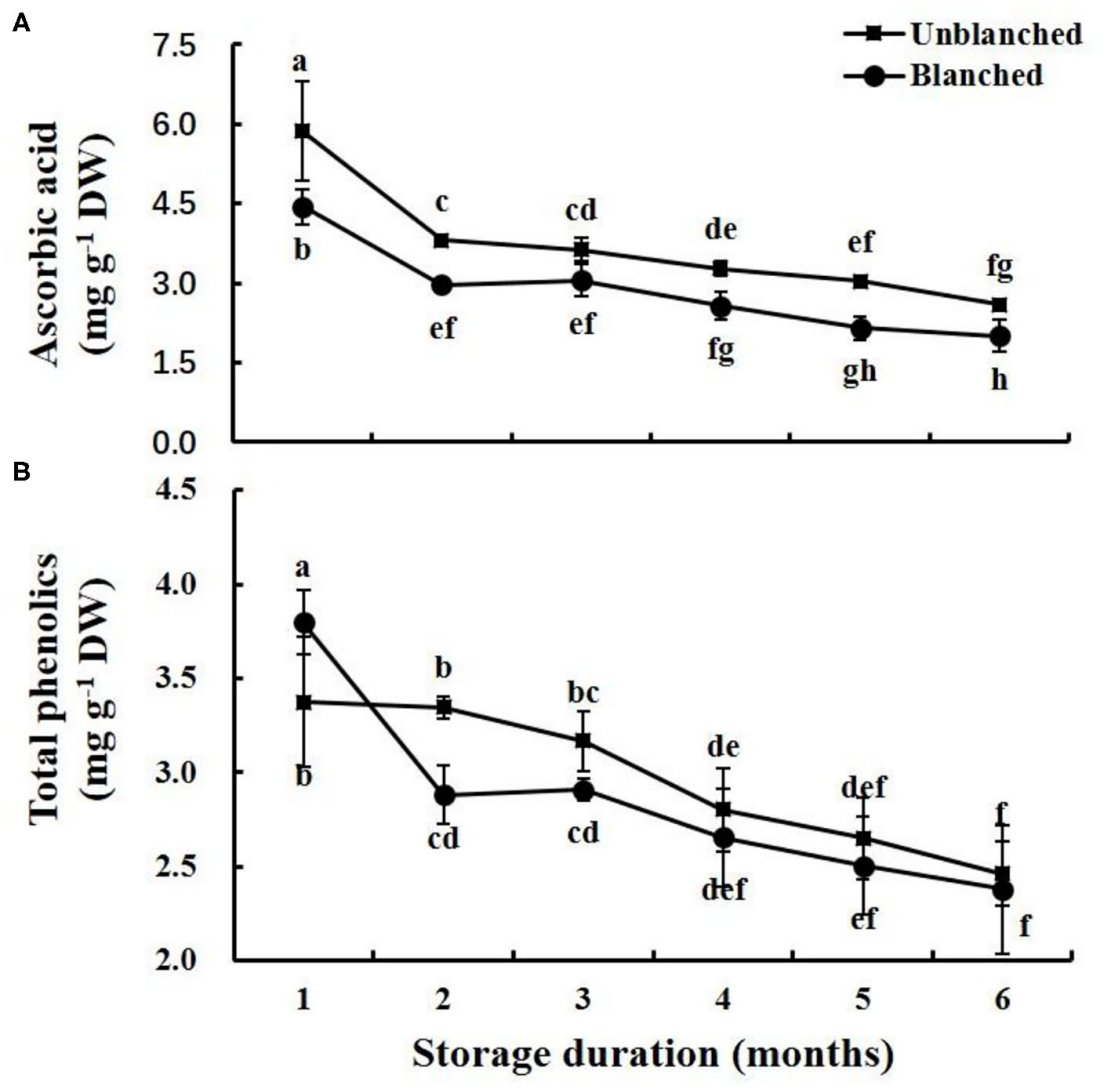
Evolution trend of ascorbic acid and total phenolics contents in baby mustard lateral buds during frozen storage at −20°C. **(A)** ascorbic acid; **(B)** total phenolics. Unblanched represents no blanching before freezing; Blanched represents blanching before freezing. Values not sharing the same letter are significantly different at *p* < 0.05.

Blanching before freezing significantly reduced the ascorbic acid content of lateral buds of baby mustard (by 24.23%), whereas it significantly increased their total phenolics content (by 12.76%) (Figure 3). During frozen storage, ascorbic acid contents in lateral buds decreased rapidly in the 1st month and then slowly went down whether the buds were blanched or not (Figure 3A). The total phenolics content in the blanched treatment group decreased rapidly in the 1st month and then slowly went down, while that in the unblanched treatment group remained stable in the 1st month and then slowly went down (Figure 3B). The ascorbic acid content in the unblanched treatment group was significantly higher than that in the blanched treatment group during storage, while total phenolics content was not significantly different between the unblanched and blanched treatment groups after 1 month. In addition, baby mustard still retained relatively high levels of ascorbic acid and total phenolics after 8 months of frozen storage, retaining 44.37% of ascorbic acid content and 73.00% of total phenolics content in the unblanched treatment group, and 34.13% of ascorbic acid content and 70.62% of total phenolics content in the blanched treatment group (Figure 3).

### Antioxidant Capacity

**Figure 4:**
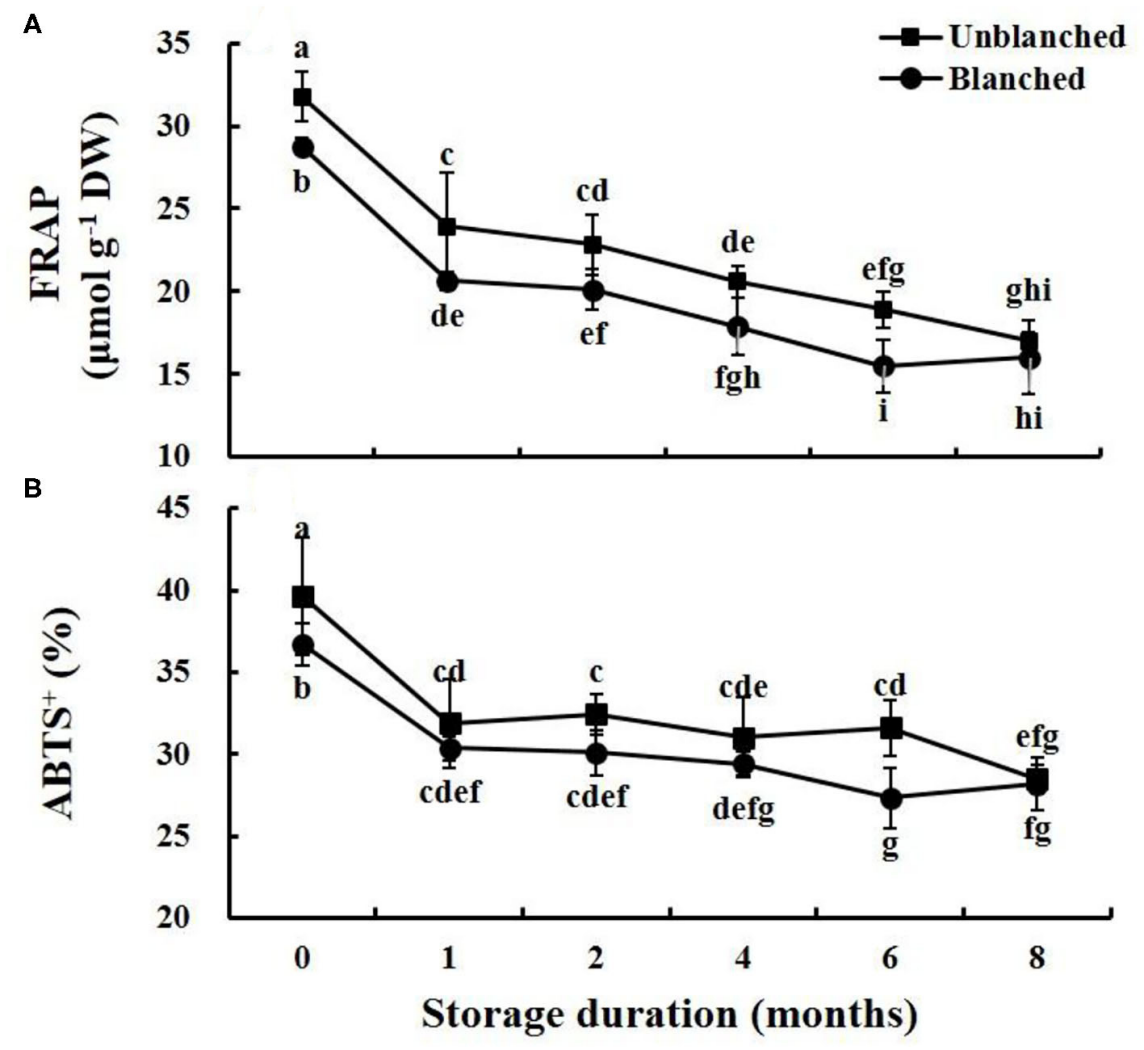
Evolution trend of antioxidant capacity levels in baby mustard lateral buds during frozen storage at −20°C. **(A)** FRAP; **(B)** ABTS^+^. Unblanched represents no blanching before freezing; Blanched represents blanching before freezing. Values not sharing the same letter are significantly different at *p* < 0.05.

We investigated antioxidant capacity using both FRAP and ABTS assays (Figure 4). The trends of antioxidant levels were similar to that of ascorbic acid content during frozen storage. Blanching before freezing significantly reduced the levels detected by both FRAP and ABTS, by 9.75 and 7.39%, respectively. During long-term frozen storage, FRAP and ABTS levels in lateral buds decreased rapidly in the 1st month and then slowly went down, while the baby mustard still retained more than 50 and 70% of its antioxidant capacity as measured by FRAP and ABTS at the end of frozen storage, whether it was blanched or not. Interestingly, the FRAP level in the unblanched treatment group was significantly higher than that in the blanched treatment group during storage except for the 8th month, while the ABTS level was not significantly different between the unblanched and blanched treatment groups except for the 6th month.

### Soluble Sugars

**Figure 5:**
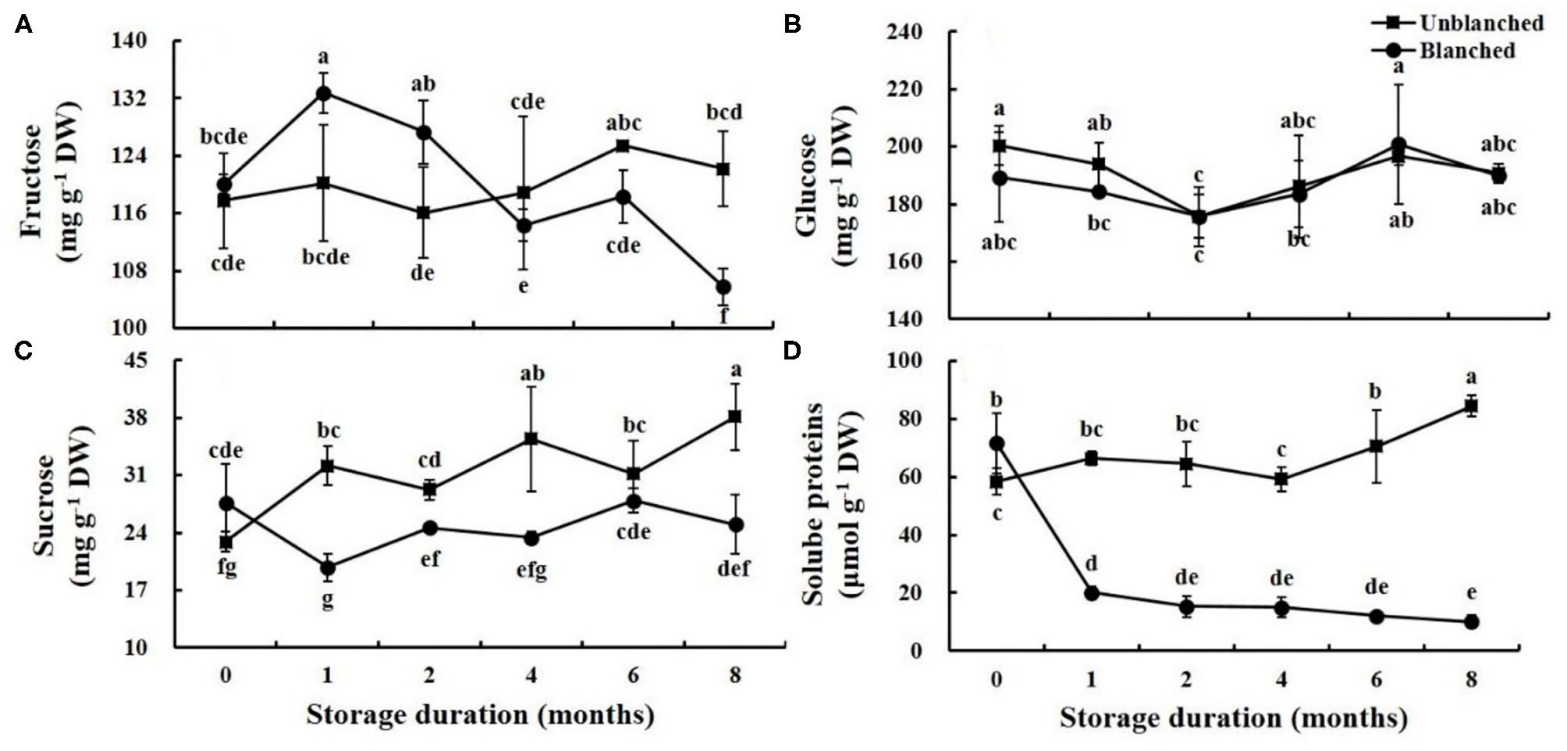
Evolution trend of soluble sugars and soluble proteins contents in baby mustard lateral buds during frozen storage at −20°C. **(A)** fructose; **(B)** glucose; **(C)** sucrose; **(D)** soluble proteins. Unblanched represents no blanching before freezing; Blanched represents blanching before freezing. Values not sharing the same letter are significantly different at *p* < 0.05.

There were three kinds of soluble sugars (fructose, glucose, and sucrose) detected in lateral buds of baby mustard (Figures 5A–C). Blanching had no significant effect on the fructose and glucose contents, whereas it significantly increased sucrose content. During long-term frozen storage, the fructose content of lateral buds increased first and then decreased; peak fructose content appeared early and high in the blanched treatment group compared with the unblanched treatment group (Figure 5A). Both glucose contents and the change trends were similar between the unblanched and blanched treatment groups, first decreasing and then increasing (Figure 5B). The trend of sucrose content in the blanched treatment group was similar to that of glucose, while the sucrose content of the unblanched treatment group showed a wavelike enhancement (Figure 5C).

### Soluble Proteins

Blanching before freezing significantly increased soluble protein content in lateral buds of baby mustard (by 22.55%). In long-term frozen storage, the soluble protein content in the blanched treatment group dropped dramatically (by 71.91%) in the 1st month, and then remained stable. However, soluble protein content in the unblanched treatment group remained basically stable for the first 4 months, and then increased significantly (by 42.49%) (Figure 5D).

### A Time-Related Trajectory Analysis

**Figure 6:**
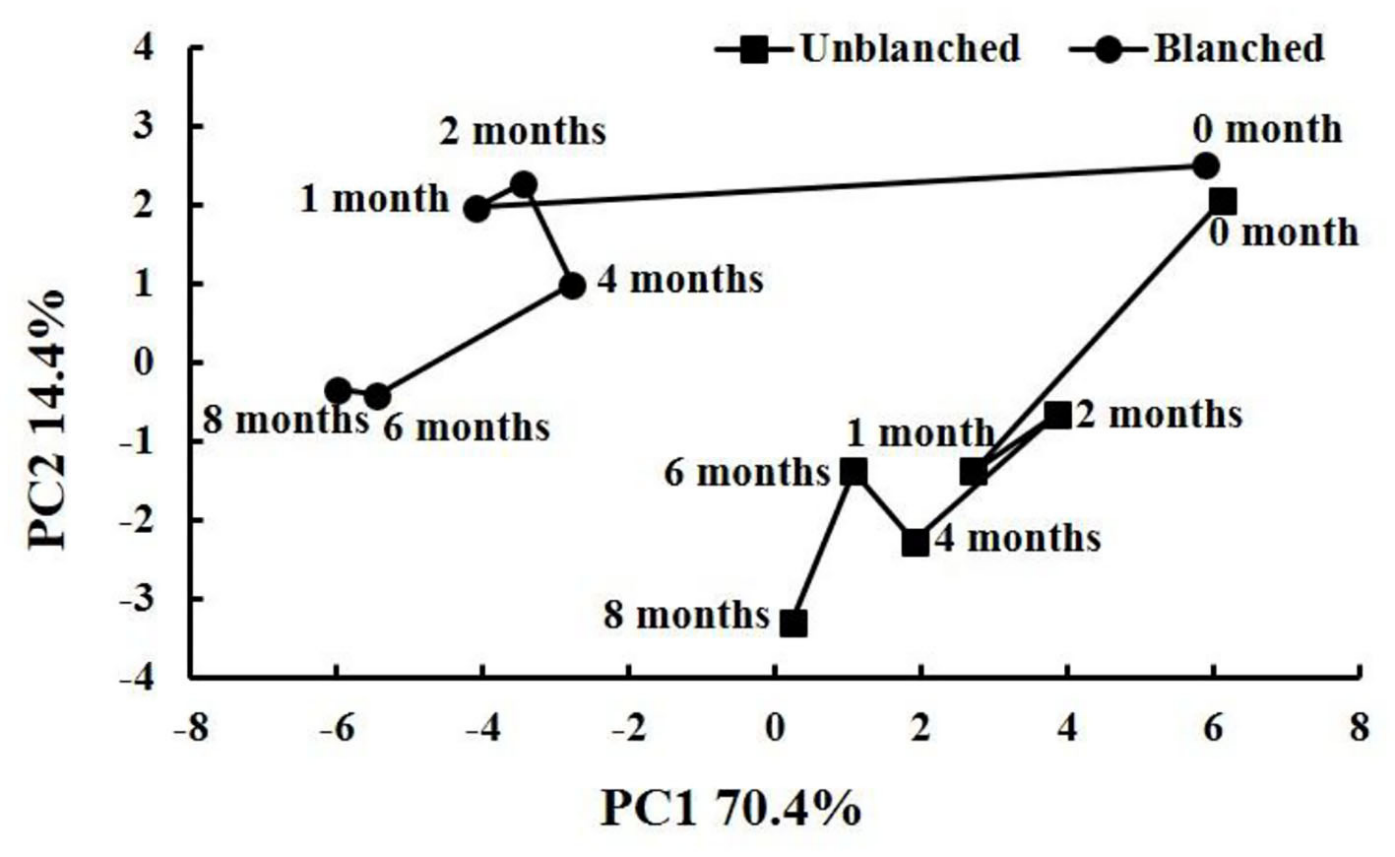
Time-related trajectory plot showing the dynamic time-related responses of nutritional qualities in baby mustard lateral buds during frozen storage at −20°C. Unblanched represents no blanching before freezing; Blanched represents blanching before freezing.

A time-related trajectory analysis was performed to compare the impacts of long-term frozen storage and blanching before freezing on the retention of health-promoting phytochemicals and antioxidant capacity in baby mustard (Figure 6). The points representing different storage times and blanching status before freezing were notably separated, and the greater the distance from the origin (month 0), the higher the degree of deterioration. During long-term frozen storage, both unblanched and blanched treatment groups had the largest distance change in the 1st month and little change thereafter. In general, the unblanched treatment group had a shorter distance change than the blanched treatment group throughout storage. Thus, the loss of nutritional qualities mainly occurred in the 1st month of frozen storage, and the nutritional qualities in the unblanched treatment group were significantly better than those in the blanched treatment group during long-term frozen storage.

## Discussion

Glucosinolates are important bioactive compounds in baby mustard which have potential anticancer activity through inhibiting tumor cell growth (6, 19, 20). Previous studies have shown that glucosinolate content in *Brassica* vegetables (including broccoli, cauliflower, and Brussels sprouts) may be effectively maintained by long-term frozen storage (12, 15, 21). Similar results were also achieved in this study. After 8 months of long-term frozen storage, ~50% or more of the total glucosinolate content of baby mustard was retained in both unblanched and blanched treatment groups. However, we found in a previous study that the total glucosinolate content of baby mustard was reduced by more than 80% after 6 days of storage at room temperature (20 ± 2°C) in the dark (6). This difference may be due to the inactivation of myrosinase activity caused by low temperature, which slowed down the degradation of glucosinolates to minimal rates during freezing (22). The loss of glucosinolate contents mainly occurred in the first month of frozen storage, which may be related to disruption of plant cells by ice crystals during freezing (15). However, the loss of glucosinolates in the blanched treatment group in the first month of frozen storage was greater than that in the unblanched treatment group. This is probably due to blanching leading to increased solute loss, which raises the freezing point, and therefore an increase in the amount and size of ice crystals (14). Furthermore, blanching before freezing resulted in a decline in high-content glucosinolate levels (Figures 1A,C), but either had no effect on low-content glucosinolates or even increased content (Figures 1B,D–F and Supplementary Figure 1A). According to previous reports, remarkable reductions in high-content glucosinolates may be explained by the dissolution of glucosinolates into water as cell lysis occurs and glucosinolates degrade during blanching (21, 23). Similar results have also been found in other *Brassica* vegetables, including broccoli florets, brussels sprouts, cauliflower, and curly kale (15, 21). On the other hand, for low-content glucosinolates, one possible explanation is that the effects of blanching through deactivation of myrosinase (15, 21, 22) and improvement of the extractability of glucosinolates (11) balanced or exceeded the effects that would normally reduce glucosinolates.

Vegetable color is an important factor in terms of quality judgment by consumers (4, 24). The color of lateral buds of baby mustard is mainly determined by chlorophylls and carotenoids (6, 17). In this study, pigment contents in the unblanched treatment group did not change significantly during frozen storage, which may be due to cell structure remaining intact without solute loss before freezing; low temperature then substantially reduced metabolic processes and inhibited the degradation of pigments (10). The change of pigment contents in the blanched treatment group was similar to of the change in glucosinolate contents, which remained stable after a sharp decrease in the 1st month of frozen storage. This pigment loss may be due to solute loss, which could increase the freezing point and amount and the size of ice crystals (14), resulting in reduced pigment contents. However, due to inactivation of related enzyme activity during frozen storage, more than 80% of carotenoid content and approximately half of chlorophyll content remained in the blanched treatment group after 8 months of long-term frozen storage. On the whole, these findings indicate that frozen storage is an excellent way to preserve pigment contents in baby mustard. Interestingly, blanching before freezing did not have a significant effect on pigment contents, in contrast to glucosinolate contents (Figure 1). A similar result was also found in broccoli (21), which may be due to pigment enzyme activity being blocked during blanching (21), fat-soluble nature pigments being less prone to leaching effects (25), and even to the fact that blanching increases the extractability of pigments (11).

Antioxidants can provide electrons or hydrogen atoms, participate in the scavenging of free radicals, and prevent the accumulation of free radicals in vegetables (8, 26). Long-term frozen storage is an effective way to reserve the ascorbic acid in frozen baby mustard, and we found that retention of ascorbic acid in both unblanched and blanched treatment groups was still ~40% after 8 months of frozen storage. This can be explained by the inactivation of oxidase at low temperature (16). Ascorbic acid is highly water-soluble and sensitive to heat, and these properties make it susceptible to blanching (27, 28), causing ascorbic acid content in baby mustard to decrease by more than one-fifth through blanching before freezing. In addition, according to the previous literature, ascorbic acid also continues to degrade during prolonged storage of frozen products (28); our results were consistent with this finding. Likewise, long-term frozen storage is also an effective way to preserve the total phenolics in frozen baby mustard. After 8 months of long-term frozen storage, over 70% of total phenolics contents were still retained in both unblanched and blanched treatment groups. Unlike ascorbic acid, total phenolics content in baby mustard significantly increases from blanching before freezing. It may be that the blanching process inactivates enzyme activity that causes the oxidation of phenolics (28), leading to the enhancement of total phenolics content after blanching. In our study, phenolic degradation still occurred in baby mustard during frozen storage, which was consistent with previous results on raspberries and blackberries, with 12 and 8% loss of total phenolics, respectively (29). Antioxidant capacity reflects the synergetic effect of multiple antioxidants (21, 30). In this study, the change trend of antioxidant capacity is similar to the change trend of ascorbic acid content, suggesting that ascorbic acid may contribute more to antioxidant capacity in baby mustard than phenolic compounds, which agrees with our previous results (6).

Soluble sugars and soluble proteins are important quality indicators for horticultural crops (17, 31). In this study, baby mustard still retained relatively high levels of soluble sugars content after 8 months of long-term frozen storage. Environmental stress during postharvest storage can induce sucrose content (32). Our results found that sucrose content in the unblanched treatment group significantly increased during frozen storage, while that in the blanched treatment group was basically stable. A possible reason is that blanching before freezing could lead to inactivation of enzymes responsible for sucrose metabolism (33), while the unblanched treatment group still had a certain level of activity of sucrose biosynthetic enzymes at low temperature (14). For soluble proteins in baby mustard, blanching before freezing significantly increased content, which may be due to the rapid production of a large number of heat shock proteins and proline in the bud cells (34, 35). Subsequently, soluble proteins content in the blanched treatment group decreased rapidly in the 1st month, which may be explained by an increase in the number and volume of ice crystals formed during the freezing process and caused by the blanching process (14).

In this study, it was observed that the main health-promoting compounds such as glucosinolates, carotenoids, and ascorbic acid, as well as antioxidant capacity in baby mustard deteriorated to varying degrees during long-term frozen storage and the blanching process, indicating that related enzymes may play an important role in regulation of these metabolites during blanching and frozen storage. Previous studies reported that freezing can inactivate peroxidase and lipoxygenase, thus improving the quality of frozen food (36), while blanching process could cause an almost complete loss of myrosinase activity, which subsequently resulted in the inhibition of glucosinolate hydrolysis (21). The effect of enzymes on postharvest storage of baby mustard is interesting, and therefore it would be included in our future research.

## Conclusion

In summary, frozen storage successfully maintained the health-promoting compounds and antioxidant capacity in baby mustard for a long time, which could make year-round supply of baby mustard possible. We recommend against blanching baby mustard before frozen storage, in order to preserve high nutritional quality. However, further experiments are still needed to find ways to improve the quality of baby mustard during long-term frozen storage, including blanching technology improvements and application of new quick-freezing technologies.

## Data Availability Statement

The original contributions presented in the study are included in the article/Supplementary Material, further inquiries can be directed to the corresponding author/s.

## Author Contributions

HL and BS designed the experiments. JZ, HD, PX, ZW, and ZL conducted the experiments. FZ, CZ, SH, ML, YT, and YL analyzed the data. JZ and HD wrote the manuscript. FZ and BS revised the manuscript. FZ, HL, and BS provided the financial support. All authors have read and agreed to the published version of the manuscript.

## Conflict of Interest

The authors declare that this study received funding from Zhishi Supply Chain Technology Co., Ltd. The funder was not involved in the study design, collection, analysis, interpretation of data, the writing of this article or the decision to submit it for publication.

## Supplementary Material

Supplementary Figure 1 Evolution trend of 4-hydroxy glucobrassicin and total glucosinolate contents in baby mustard lateral buds during frozen storage at −20°C. Unblanched represents no blanching before freezing; Blanched represents blanching before freezing. Values not sharing the same letter are significantly different at *p* < 0.05.
