# Supplementary material for: Effect of Long-Term Frozen Storage on Health-Promoting Compounds and Antioxidant Capacity in Baby Mustard
Source: Front Nutr. 2021 Apr 6;8:665482. doi: 10.3389/fnut.2021.665482 (PMC8055821; doi:10.3389/fnut.2021.665482)
Supplement: Supplementary Figure 1 — Evolution trend of 4-hydroxy glucobrassicin and total glucosinolate contents in baby mustard lateral buds during frozen storage at −20°C. Unblanched represents no blanching before freezing; Blanched represents blanching before freezing. Values not sharing the same letter are significantly different at p < 0.05. [file Data_Sheet_1.docx]

Supplementary Material

## Supplementary Figure 1


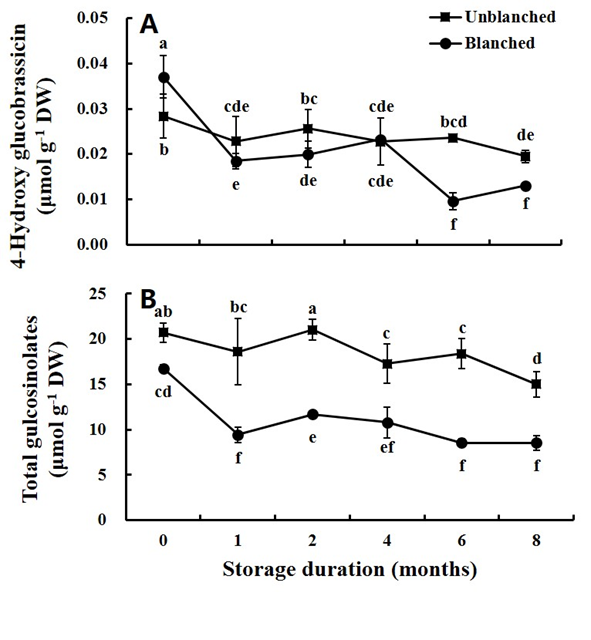


**Supplementary Figure 1.** Evolution trend of 4-hydroxy glucobrassicin (A) and total glucosinolate contents (B) in baby mustard lateral buds during frozen storage at -20 °C. Unblanched represents no blanching before freezing; Blanched represents blanching before freezing. Values not sharing the same letter are significantly different at *p*<0.05.
